# The Epidermal Keratinocyte as a Therapeutic Target for Management of Diabetic Wounds

**DOI:** 10.3390/ijms24054290

**Published:** 2023-02-21

**Authors:** Wei-Cheng Fang, Cheng-Che E. Lan

**Affiliations:** 1 Department of Dermatology, Kaohsiung Medical University Hospital, Kaohsiung Medical University, Kaohsiung 80708, Taiwan; 2 Department of Dermatology, College of Medicine, Kaohsiung Medical University, Kaohsiung 80708, Taiwan

**Keywords:** diabetes mellitus, keratinocyte, diabetic wound healing

## Abstract

Diabetes mellitus (DM) is an important cause of chronic wounds and non-traumatic amputation. The prevalence and number of cases of diabetic mellitus are increasing worldwide. Keratinocytes, the outermost layer of the epidermis, play an important role in wound healing. A high glucose environment may disrupt the physiologic functions of keratinocytes, resulting in prolonged inflammation, impaired proliferation, and the migration of keratinocytes and impaired angiogenesis. This review provides an overview of keratinocyte dysfunctions in a high glucose environment. Effective and safe therapeutic approaches for promoting diabetic wound healing can be developed if molecular mechanisms responsible for keratinocyte dysfunction in high glucose environments are elucidated.

## 1. Introduction

Diabetes mellitus (DM), an important global health issue, is a metabolic disease characterized by impairment in regulating glucose homeostasis. The total number of diabetic patients is expected to increase from 171 million in 2000 to 366 million in 2030 [1]. A hyperglycemic state ultimately leads to the development of macrovascular and/or microvascular complications involving the eyes, kidneys, nerves, heart, and blood vessels [2]. Poor diabetic wound healing is one of the major complications of DM patients, leading to ulceration, infection, and ultimately amputation [3]. The incidence of foot ulcers in DM patients has been estimated to be 19 to 34% [4]. They remain a primary cause of morbidity and mortality in patients with diabetes [5]. Due to the increasing prevalence of diabetes worldwide, uncovering the underlying molecular mechanisms that are responsible for the poor wound healing of DM patients is a vital public health issue that needs to be addressed. Wound healing is a complicated multicellular process that includes coagulation, inflammation, proliferation, and remodeling phases. Platelets, inflammatory cells, fibroblasts, and endothelial cells have been known to play an important role in the wound healing process. In recent years, the key role of keratinocyte in wound healing has been investigated [6,7]. Keratinocytes can cover wound surfaces to regenerate an epithelial barrier with the outside environment. Keratinocytes secrete multiple cytokines to stimulate re-epithelialization, angiogenesis, and the production of a connective tissue matrix. Furthermore, keratinocytes are at the frontlines of innate immunity. After injury and the invasion of microorganisms, keratinocytes release various cytokines, chemokines, and antimicrobial peptides (AMPs) which activate immune cells and eliminate pathogens directly [8]. However, diabetic wounds have a microenvironment with hyperglycemia, advanced glycation end products (AGEs), mitochondrial dysfunction, reactive oxygen species (ROS), and inflammatory cytokines that may contribute to the impairment of keratinocyte functions. Physiological dysfunctions of keratinocytes in high glucose environments include prolonged inflammation, impaired proliferation, and migration ability, resulting in delayed wound healing. Herein, it is important to investigate the physiological functions and molecular mechanisms of keratinocytes in diabetic wound healing. The current standard treatment for diabetic foot ulcers includes surgical debridement, anti-infection treatments, wound dressing, pressure off-loading, and vascular surgery [9]. However, long-term surgical intervention and repeat dressings will cause severe pain and economic burden to the patients. Therapeutic approaches targeting the epidermal keratinocyte may bring new hope for optimal diabetic wound care.

## 2. Normal Wound Healing

Wound healing is a complex multicellular process involving platelets, neutrophils, and macrophages, fibroblasts, endothelial cells, and keratinocytes. It follows four stages—the coagulation, inflammation, proliferation, and remodeling phases [10].

### 2.1. Coagulation

Coagulation is the first step of wound healing leading to clot formation and activation of the intrinsic and extrinsic coagulation cascade. Immediately after injury, vasoconstriction contributes to the reduction of bleeding and is followed by the accumulation and activation of platelets. Activated platelets release growth factors in alpha granules including platelet-derived growth factors (PDGF), insulin-like growth factors (IGF), epidermal growth factors (EGF), transforming growth factor-β (TGF-β), and platelet factor 4 [11,12,13] to recruit other platelets and inflammatory cells, and promote the proliferation and migration of fibroblasts and endothelial cells to the injury site [14]. The intrinsic and extrinsic coagulation cascades are initiated and result in the transformation of prothrombin into thrombin. Thrombin then catalyzes the conversion of fibrinogen to fibrin and activates Factor XIII. Activated Factor XIII functions to crosslink fibrin chains, leading to the clot formation that acts as a matrix for cell migration.

### 2.2. Inflammation

Inflammation begins within 24 to 48 h after injury, and the characteristic of this phase is migration of inflammatory cells to the injury site. Neutrophils, the first arrived inflammatory cells, adhere to the vascular endothelium and further migrate into the extravascular space. Neutrophils have multiple functions, including antimicrobial ability and the production of proinflammatory cytokines, enzymes and oxygen-derived free radicals [15]. Macrophages typically appear within 72 h after injury. Macrophages are the most important regulatory cells in the inflammatory phase for the phagocytosis of necrotic material and bacteria, releasing proteolytic enzymes and growth factors for extracellular matrix definition (ECM) production, including platelet-derived growth factor, fibroblast growth factors (FGFs), and vascular endothelial growth factors (VEGFs), as well as TGF-β and TGF-α. Macrophages can be divided into M1 (classically activated) and M2 (alternatively activated) macrophages [16,17]. M1 macrophages, activated by interferon-γ (IFN-γ) and TNF-α, are represented as a pro-inflammatory phenotype, showing increased phagocytic and antigen presenting capacities, pro-inflammatory cytokine and oxidative metabolite production to promote host defense, and the elimination of necrotic tissues [18,19]. On the other hand, M2 macrophages demonstrate a phenotype in the resolution of inflammation by releasing anti-inflammatory cytokines such as IL-10 [20]. M2 macrophages are derived from resting macrophages after exposure to Th2 cytokines, such as IL-4 or IL-13 [21]. They arrive later in the wound healing process for granulation tissue formation. Notably, this M1/M2 terminology is determined based on in vitro experiments [22,23], and has been challenged by in vivo studies [24,25,26]. Actually, macrophages can coexpress both M1 and M2 markers during different stages of wound healing [27,28]. Using a small number of markers to categorize M1 or M2 macrophages is not accurate. Pang et al. used single cell RNA-sequencing and downstream analysis to reveal the different phenotypes and transitions of macrophages in the course of wound healing in mice [29].

### 2.3. Proliferation

The proliferative phase is characterized by fibroblast migration, ECM deposition, granulation tissue formation, neovascularization, and re-epithelialization. Fibroblasts are attracted by PDGF and TGF-β and produce components of ECM, including fibronectin, hyaluronan, collagen and proteoglycans. The formation of ECM is crucial for tissue repair and serves as a scaffold for cell growth and migration [30]. The main structural element of the ECM is collagen. The synthesis of collagen is stimulated by PDGF, basic FGF (bFGF), TGF-β, IL-1, and TNF. Integrins are transmembrane proteins binding the ECM to cytoskeletal structures and are important in cell–cell and cell–matrix adhesion [31]. M2 macrophages in this stage produce anti-inflammatory cytokines, VEGFs and TGF-β for induction of cell proliferation and the granulation of tissue formation [9]. Neovascularization, also a characteristic of this stage, is stimulated by different angiogenic factors, including VEGF and fibroblast growth factor-2 (FGF-2) secreted from keratinocytes, fibroblasts and inflammatory cells [32]. α3β1 integrin in keratinocytes induces the secretion of proangiogenic factors that promotes endothelial-cell migration leading to angiogenesis [33]. Re-epithelialization is a critical event in the proliferative phase and is regulated by the migration and proliferation of keratinocytes from the wound edges or skin adnexal structures [34,35]. Re-epithelialization is induced by growth factors such as the endothelial growth factor (EGF), the keratinocyte growth factor (KGF), and the FGF-2 secreted from keratinocytes and other cells [9]. During keratinocyte migration, matrix metalloproteinases (MMPs) are important for the detachment of keratinocytes from the hemidesmosome and desmosome. MMP-1 can bind the α2β1 integrin upon release from keratinocytes migrating on type I collagen [11]. MMP-9 plays a crucial role in breaking down Type IV and Type VII collagen, which are major components of the anchoring fibrils and basement membrane [36]. After breaking down these complicated structures that anchor the keratinocytes to the basement membrane and nearby keratinocytes, keratinocyte migration begins and is important for the resurfacing of the wound. In the normal tissue, MMP-9 is expressed at a low level, and is upregulated in wounds. As the wound heals, MMP-9 is downregulated [37]. However, the persistent expression of MMP-9 in chronic wounds contributes to impaired wound healing. The balance of the bimodal expression of MMP-9 is important to the epithelialization.

### 2.4. Remodeling

Remodeling, the final phase of wound healing, occurs around 2–3 weeks after injury and may continue for months. During this phase, the granulation tissue is gradually replaced by mature scar tissue [38]. The remodeling of collagen including the synthesizing of new collagen and collagen degradation is mediated by fibroblasts and MMPs. Collagen type III is gradually replaced by collagen type I, which has greater tensile strength [39]. Fibroblasts interact with ECM, leading to wound contraction, which is influenced by multiple cytokines, including TGF-β, PDGF, and bFGF. The phenotypic switch from fibroblasts to myofibroblasts promotes wound contraction, leading to potential scar formation, which is induced by keratinocytes through TGF-β signals [40,41,42].

## 3. Functional Impairments of Keratinocytes in Chronic Diabetic Wounds

**Figure 1:**
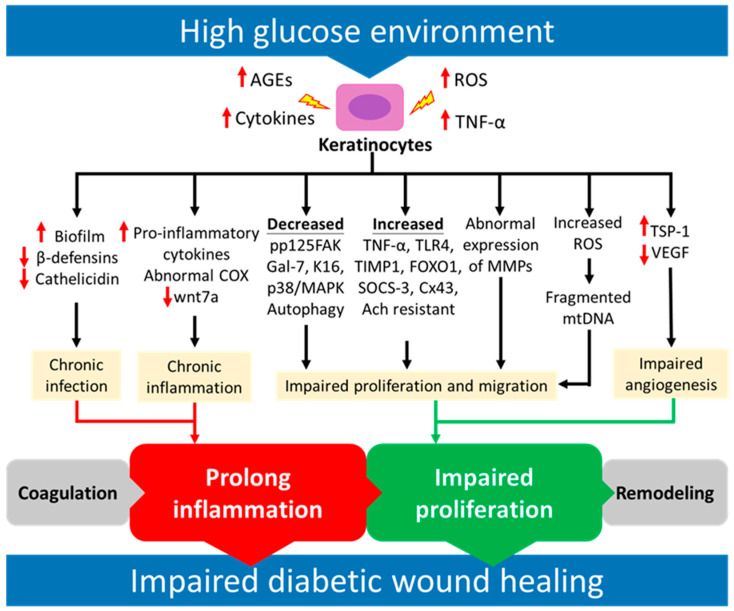
Scheme revealing different factors associated with dysfunctional keratinocytes in diabetic wound healing. Advanced glycation end products (AGEs), reactive oxygen species (ROS), and inflammatory cytokines may contribute to the impairment of keratinocyte migration and proliferation, chronic inflammation, chronic infection, and impaired angiogenesis in high glucose environments, leading to impaired diabetic wound healing due to prolonged inflammation and the impaired proliferation phase of wound healing.

Diabetes is a metabolic disease characterized by hyperglycemia, and is a major cause of chronic wounds that may lead to amputation in affected patients. Diabetic wounds have a microenvironment with elevated levels of glucose, advanced glycation end products (AGEs), mitochondrial dysfunction, reactive oxygen species (ROS), and inflammatory cytokines that may contribute to the impairment of keratinocyte migration and proliferation, chronic inflammation, chronic infection, and impaired angiogenesis (Figure 1).

### 3.1. Increased Oxidative Stress in Keratinocytes

Emerging evidence has shown that the hyperglycemic environment can increase oxidative stress, which indicates an imbalance between free radical formation and adequate antioxidant capacity [43,44]. The increased ROS level may contribute to the impairment of the ability for wound healing through altering the mitochondrial membrane potential, mass, and morphology in mononuclear cells of diabetic patients [45,46] and increasing TNF-α in mouse models [47]. ROS can also decrease the diversity of the skin microbiota that promotes biofilm formation and further prolongs wound healing [48]. Our previous study showed that elevated ROS levels in a high-glucose environment contribute to the increase in IL-8 production from keratinocytes, and neutrophil infiltration results in impaired wound healing in a diabetic rat model [49]. ROS can upregulate MMP-9 through the activation of nuclear factor kappa beta (NF-κB) in human keratinocytes, leading to the impairment of keratinocyte migration [50,51]. In addition, the mitochondria, a main source of ROS production, can generate huge amounts of ROS in a high glucose environment, followed by the hampering of the antioxidant ability of the cell and resulting in mitochondria damage [52,53]. Excessive ROS then causes the loss of mitochondrial membrane potential and further mtDNA fragmentation. The fragmented mtDNA translocate into to cytosol and involve cGAS-STING-IRF3 activation via the ERK1/2-PI3K/Akt-tuberin-mTOR pathways [54,55]. Activated interferon regulatory factor 3 (IRF3) then promotes the inflammatory reaction and triggers keratinocyte apoptosis [56].

### 3.2. Abnormal Expression of Matrix Metalloproteinases (MMPs)

MMPs are endopeptidases involved in degrading extracellular matrix elements such as collagen, fibronectin and laminin, and have been revealed to play critical roles in wound healing due to influencing keratinocyte migration. The activities of MMPs are mediated by the tissue inhibitors of MMPs (TIMPs), and the abnormal expression of MMPs and TIMPs have been linked to delayed wound healing in diabetes. Our previous work revealed that a high glucose environment suppressed keratinocyte migration, reduced mRNA levels and the activity of MMP-2 and MMP-9, but increased the expression of TIMP-1 in cultured keratinocyte [57]. We also demonstrated that keratinocyte cultured in a high glucose environment decreased the expression of MMP-1 and α2β1 integrin, which are crucial for the migration of keratinocytes on type I collagen. These events contribute to delayed diabetic wound healing [58]. Additionally, keratinocyte derived MMPs may be mediated by cytokines produced by circulating mononuclear cells. Our previous study showed that the decreased expression of IL-22 from peripheral blood mononuclear cells may suppress the production of MMP-3 in cultured keratinocytes and the wounds of diabetic rats, leading to impaired keratinocyte migration in high glucose environments [59]. Chang et al. revealed that infected diabetic wounds increase active MMP-9, increases inflammation, and decreases angiogenesis leading to prolonged wound healing. (R)-ND-336, a potent and selective inhibitor of MMP-9, can promote the healing of infected diabetic wounds in a mouse model [60].

### 3.3. Impaired Proliferation and Migration of Keratinocyte

**Figure 2:**
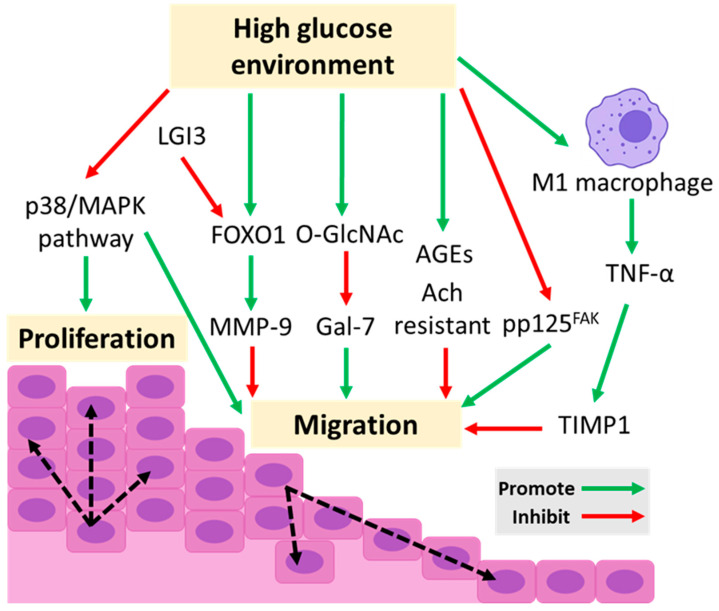
Factors influencing the proliferation and migration ability of keratinocytes in a high glucose environment. The high glucose environment downregulates the expression of phosphorylated p125^FAK^ (pp125^FAK^) and the p38/mitogen-activated protein kinase (MAPK) pathway, leading to the inhibition of keratinocyte migration. Increased O-linked N-acetylglucosamine (O-GlcNAc) glycosylation in a high glucose environment is responsible for reduced Gal-7 expression, which plays an important role in keratinocyte migration. Increased forkhead box protein O1 (FOXO1) in keratinocytes can stimulate the expression of MMP-9, leading to the impairment of keratinocyte migration. Leucine-rich repeat LGI family member 3 (LGI3) can promote the degradation of FOXO1 through phosphorylation of FOXO1 via the Akt pathway. A high glucose environment stimulates the formation of advanced glycation end products (AGEs) and keratinocyte resistance to acetylcholine (Ach), leading to the impairment of keratinocyte migration. A high glucose environment induces M1 macrophage infiltration, followed by the increased secretion of TNF-α, which upregulates tissue inhibitor matrix metalloproteinase 1 (TIMP-1) expression in keratinocytes, resulting in impaired keratinocyte migration.

Keratinocyte migration is important in the re-epithelialization stage of wound healing. Our previous study revealed that a high glucose environment downregulated the expression of phosphorylated p125^FAK^ (pp125^FAK^) in cultured human keratinocytes, which is a crucial factor in the organization of cytoskeletal protein and cell migration [57]. The hyperglycemic environment promotes the polyol pathway, resulting in increasing intracellular sorbitol and further stimulating the formation of AGEs and pro-inflammatory cytokines. Keratinocytes cultured with AGE modified human serum albumin showed impairment of keratinocyte adhesion and migration as well as the decreasing expression of integrin alpha 3 [61]. In addition, increased O-linked N-acetylglucosamine (O-GlcNAc) glycosylation in a high glucose environment is responsible for reduced Gal-7 expression in cultured human keratinocytes, which plays an important role in keratinocyte migration [62]. p38/mitogen-activated protein kinase (MAPK) is also an important kinase promoting keratinocyte migration and proliferation through the reorganization of the cytoskeleton [63,64,65,66]. Autophagy, a downstream target of the p38/MAPK pathway for the degradation of misfolded proteins [67,68,69], has been revealed as a regulator in early differentiation [70,71], cell death [72,73], and the cell migration of keratinocytes [74,75]. Li et al. demonstrated that the p38/MAPK pathway in human immortalized keratinocyte HaCaT cells is downregulated and followed by the inactivation of autophagy in a high glucose environment, leading to the impairment of keratinocyte migration [76]. The migration of keratinocytes from the perilesional area is essential for re-epithelialization. Our previous study revealed that an increased percentage of M1 macrophages and a high level of TNF-α were detected in the perilesional area of diabetic rats. We further found that a high glucose environment induces M1 macrophage infiltration followed by the increased secretion of TNF-α, which upregulates the TIMP-1 expression in keratinocytes, resulting in impaired keratinocyte migration. The recovery rate of a wound can be significantly improved after the administration of a TNF-α inhibitor to the perilesional area of diabetic rats [77]. Leucine-rich repeat LGI family member 3 (LGI3) has various functions involved in neuronal exocytosis, β-amyloid endocytosis, and it induces neuronal differentiation. A recent study found that the increasing expression of LGI3 can restore cell migration in a high glucose environment and reduce LGI3 expression by siRNA into HaCaT cells, inhibiting wound closure [78]. KIM et al. revealed that LGI3 in HaCaT cells can regulate the migration of keratinocytes via the Akt pathway, which also plays an important role in keratinocyte migration and differentiation, influencing wound healing ability [79] through the phosphorylation of forkhead box protein O1 (FOXO1) [80] and β-catenin [81]. Recent studies revealed that increased FOXO1 in keratinocytes can diminish the expression of TGF-β1 but stimulate the expression of MMP-9, CCL20, IL-36γ, and SERPINB2, leading to the impairment of re-epithelialization, connective tissue healing, and angiogenesis in diabetic conditions [7]. In addition to keratinocyte migration, the proliferation of keratinocytes and the dynamic expression of gap junctions between keratinocytes are also a critical step in re-epithelialization. Previous studies have shown that decreased basal epidermal proliferation and decreased induction of keratinocyte mitogens including KGF are noted in the wound healing of diabetic mice [82,83]. The mechanism of this phenomenon is currently unclear, but may be related to the abnormal expression of apoptotic proteins [84], impaired K16 expression [58] or the increased expression of suppressors of cytokine signaling (SOCS)-3 in keratinocytes [85] in a high glucose environment. Increased Connexin 43 (Cx43) expression, a gap junction protein, has been demonstrated in keratinocytes from the wound edge in diabetic rats [86,87]. Further knockout Cx43 in mice can accelerate re-epithelialization in wound healing [88]. Acetylcholine (Ach) is not only a cholinergic neurotransmitter, but also has non-neuronal functions in the activation of cholinergic signaling in nonneuronal cells. Interestingly, keratinocytes are one of the nonneuronal cells which is responsive to Ach with an unknown role and mechanism in re-epithelialization [89,90]. A recent study demonstrated that Ach could upregulate the expression of TGFβRII in cultured human keratinocytes by activating the Src-ERK pathway to promote TGFβ1-SMAD2-mediated epithelial mesenchymal transition (EMT). However, keratinocytes in a high glucose environment were resistant to Ach due to the activation of the p38 kinase pathway, which inhibits the Src-ERK cascade leading to reduced TGFβRII, the impairment of the TGFβ1-mediated signaling pathway, and delayed EMT in diabetic mice [91]. Therefore, a high glucose environment may inhibit keratinocytes’ response to Ach and further impair diabetic wound healing. Figure 2 summarizes the factors that affect the proliferation and migration ability of the keratinocytes in a high glucose environment. 

### 3.4. Chronic Inflammation

In wound healing, the acute inflammatory phase may last 2 weeks. The prolongation of inflammation may impair wound healing. Previous studies revealed that more neutrophils and macrophages were noted in diabetic wounds [9,15,49,92]. Increased pro-inflammatory cytokines such as IL-1, IL-6, IL-8 and TNF-α were also found [9,93]. Neutrophils play a crucial role in the inflammatory phase, and it can generate ROS and serine proteases for preventing wound infections. However, prolonged neutrophil infiltration may impair wound healing. Our previous study demonstrated that reducing pro-inflammatory cytokines and decreasing neutrophil infiltration promoted diabetic wound healing in a diabetic rat model [49]. Recent studies revealed the important role of keratinocytes, which secrete various chemokines and pro-inflammatory cytokines in the chronic inflammation of diabetic wounds. We have revealed that increased IL-8 expression from keratinocytes in a high-glucose environment is known to recruit and activate neutrophils that produce ROS, contributing to impaired diabetic wound healing [49,93]. The activation of TNF-α and toll-like receptor 4 (TLR4) signaling pathways in monocytes and endothelial cells due to increased oxidative stress in the high-glucose environment have been found in diabetic patients and animal models [94]. Cheng et al. revealed the association between TNF-α and TLR4 in keratinocytes stimulated by the high-glucose environment in animal models [95]. Wang et al. revealed that Wnt family member 7A (wnt7a) in human umbilical vein endothelial cells speeds up wound healing through the promotion of angiogenesis and the amelioration of local inflammation. The decreased expression of wnt7a is noted in wounds of diabetic rats [96]. Exogenous Wnt7a can reverse the high glucose-induced TNF-α production, TNF-α related TLR4 signaling, and high glucose-induced excessive autophagy in HaCaT cells [97]. Other keratinocyte-derived cytokines involved in different mechanisms were also found. The increased expression of macrophage inflammatory protein-2 (MIP-2) and macrophage chemoattractant protein-1 (MCP1) from keratinocytes at the wound edges were noted in the diabetic wounds of mice [92]. Kampfer et al. demonstrated that decreased cyclooxygenase-1 (COX-1) expression and increased COX-2 expression in wound margin keratinocytes of diabetic mice may influence inflammatory responses due to the abnormal production of prostaglandin [98].

### 3.5. Chronic Infection

A diabetic wound is characterized by an increased risk of infection, and an infected wound may further delay wound healing because of a prolonged inflammatory phase [99,100]. Increased ROS due to chronic inflammation may reduce the diversity of microbiota and promote biofilm formation [48]. The strain-level variation of Staphylococcus aureus, one of the dominant bacteria in human skin, is correlated with delayed re-epithelialization in diabetic wounds [101,102]. Bacteria may further provoke inflammation that deteriorates the re-epithelialization process and directly influences epidermal keratinocytes, such as by increasing apoptosis and diminishing keratinocyte migration and proliferation [103]. As a defense mechanism, keratinocytes play a key role in cutaneous innate immunity through the secretion of antimicrobial peptides including human β-defensins (HBD), cathelicidins and Psoriasin for defending bacteria, fungi, and viruses. Our previous study revealed that keratinocytes cultured in a high-glucose environment show decreasing mRNA and protein levels of HBD-2 and HBD-3. The reduction of HBD-2 is mediated by AGEs and the signal transducer and activator of the transcription (STAT)-1 pathway in human umbilical vein endothelial cells [104] and reduced HBD-3 is regulated by AGEs and the inhibition of the p38/MAPK pathway in diabetic rats [105]. Apart from its antimicrobial activity, HBD-2 has been shown to induce keratinocyte proliferation, migration, and angiogenesis through stimulating the proliferation and migration of human umbilical vein endothelial cells [106]. Cathelicidin, the other keratinocyte derived antimicrobial peptide, also showed decreased expression of mRNA and protein levels in human keratinocytes cultured in a high glucose environment, leading to decreased antimicrobial protection [107,108].

### 3.6. Impaired Angiogenesis

Angiogenesis is an important step in achieving proper wound healing by the formation of blood vessels. Several angiogenic factors including VEGF, FGF, angiogenin (RNase 5), and angiopoietins (Ang1 and Ang2) were generated by endothelial cells and keratinocytes [109,110]. The defective angiogenesis in diabetes has been linked to the impaired recruitment and migration of endothelial cells and endothelial progenitor cells (EPCs) [111]. Galiano et al. showed that the administration of VEGF in diabetic wounds can enhance angiogenesis due to the increased mobilization of EPCs from the bone marrow, which have the ability to differentiate into endothelial cells [112]. Marin-Luevano et al. revealed that synthetic innate defense regulator-1018 (IDR-1018) can promote VEGF-165 (pro-angiogenic molecules) in a cultured human endothelial cell line and HaCaT cells and reduce hypoxia-induced transcription factor-1 (HIF-1) (anti-angiogenic molecules) to stimulate endothelial cell migration [113]. We previously showed that the increased expression of the angiogenesis inhibitor Thrombospondin-1 (TSP-1), is mediated by increased DNA hypomethylation at the promoter region of TSP-1 and increased oxidative stress in cultured human keratinocytes exposed to a high glucose environment. The administration of antioxidants can normalize TSP-1 expression and improve wound healing in diabetic rats [114]. These studies indicate the important role of oxidative stress-derived TSP-1 in defective angiogenesis in diabetic wounds. In addition, the impaired expression of VEGF in keratinocytes can also induce abnormal angiogenesis, leading to chronic diabetic wounds [115,116].

## 4. Novel Therapeutic Strategies for the Treatment of Diabetic Wounds

Multiple mechanisms are involved in impaired diabetic wound healing, as described above. Targeting these pathways and correcting the physiologic functions of keratinocytes may provide novel therapeutic methods to improve wound healing in diabetic patients. For example, our previous study revealed that the administration of a TNF-α inhibitor can significantly improve wound healing in diabetic rats, since increased TNF-α in the wound environment may impair keratinocyte migration [77]. The p38/MAPK pathway is known to be involved in influencing keratinocyte migration through different mechanisms. The increased expression of FOXO1 in a high glucose environment may impair re-epithelialization and angiogenesis, which are important steps in wound healing. Targeting the p38/MAPK pathway or inhibiting FOXO1 expression may be a potential adjunctive treatment for promoting diabetic wound healing. In addition, Kulkarni et al. found that topical esmolol hydrochloride (Galnobax) can improve wound healing in diabetes through pleiotropic mechanisms [117]. It can inhibit aldose reductase and the formation of sorbitol and AGEs which interfere with keratinocyte migration, induce autophagy, and modulate macrophage polarization. Esmolol hydrochloride can also induce NO production, promoting keratinocyte proliferation and angiogenesis, which is significantly reduced in diabetic wounds [118]. Moreover, it can reduce caspase-3 and upregulate B-cell lymphoma 2 (Bcl-2) to prevent necrosis of the wound bed in animal models [119]. Therefore, esmolol hydrochloride may be a new option for the treatment of diabetic ulcers. MicroRNAs (miRNAs) are endogenous noncoding small RNAs participating in cell proliferation, apoptosis, and cell differentiation through the regulation of gene and protein expression [120]. Etich et al. showed that the changing expression of miR-204 was noted during wound healing [121]. Further studies demonstrated that the overexpression of miR-204-3p in cultured human keratinocytes can increase the expression of TGF-β and Bcl-2 in a high glucose environment, promoting the proliferation and migration of keratinocytes via suppressing levels of Bax and cleaved caspase-3. These results show that the overexpression of miR-204-3p can improve the functional impairment of keratinocytes in a high glucose environment, and it may be a novel therapeutic target for the treatment of diabetic wounds in the future [122]. In recent years, nanotechnology-based diabetic foot ulcer therapies have been developed. Nanomaterials can not only deliver drugs or cytokines to the cells, but they can also remodel the microenvironment of diabetic wounds [123]. Yoon et al. used a chemokine-loaded hydrogel to promote angiogenesis, collagen deposition, and re-epithelialization [124]. Lipid nanoparticles implanted with recombinant human EGF can promote re-epithelialization through stimulating fibroblast and keratinocyte proliferation in animal models [125]. In addition to mechanism-based therapies, various clinical trials focused on cell-based products and cell-based therapies including allogeneic keratinocyte sheets [126], autologous fibroblasts and keratinocytes implants/grafts [127,128] have been developed in the treatment of diabetic wound healing. Cell therapy is a highly promising method for diabetic wound treatment, and it can correct the factors that lead to prolonged wound healing through various mechanisms [129]. Autologous and allogeneic keratinocytes transplanted to the wound can improve wound healing via increasing the expression of growth factors [130] and ECM proteins [131]. Although allogeneic keratinocytes cannot permanently remain in the wound, they can stimulate the migration and proliferation of native keratinocytes from the wound edges in chronic leg ulcers [132]. In addition, the topical application of keratinocyte sheets has shown its effectiveness in the treatment of diabetic wounds in patients [126,133]. Furthermore, mesenchymal stem cells (MSCs) have been shown to enhance angiogenesis in pre-clinical and clinical studies [134,135]. Paracrine signaling and the ability of stem cells to differentiate into specialized cells including fibroblasts, vascular endothelial cells, and keratinocytes contribute to promote angiogenesis, neovascularization, and re-epithelialization [136]. Intravenously injected MSCs can migrate to the acute wound area and differentiate into keratinocytes, endothelial cells, monocytes, and pericytes in mice [137]. Although several studies have shown that cell therapy is a potent tool for the treatment of chronic diabetic wounds, adverse effects have also been reported [138]. Major adverse events include pulmonary and renal thromboembolism, heart failure, and liver fibrosis [139,140]. Therefore, it is important to evaluate the effectiveness of treatment and patients’ safety under cell therapy.

## 5. Conclusions

Keratinocytes play an important role in wound healing. A high glucose environment can change the gene and protein expression in keratinocytes, leading to prolonged inflammation, impaired proliferation, and the migration of keratinocytes and impaired angiogenesis during wound healing (Table 1). Elucidating the precise molecular dysfunction in keratinocytes will likely result in the development of effective and safe therapeutic approaches for optimal wound healing in patients with diabetes, as topical treatments are likely to succeed if treatment targets dysfunctional keratinocytes in the high glucose environment.

## Figures and Tables

**Table 1:** Various mechanisms related to dysfunctional keratinocytes in diabetic wound healing.

Dysfunction	Mechanism	Ref.
Increased oxidative stress and ROS	Impaired antioxidant ability results in mitochondria damage	[52]
Abnormal expression of MMPs	Decreased mRNA level and activity of MMP-2 and MMP-9 and increased TIMP-1	[57]
	Decreased expression of MMP-1 and α2β1 integrin	[58]
	Decreased expression of IL-22 may suppress production of MMP-3 in keratinocytes	[59]
Impaired proliferation and migration of KCs	Decreased expression of phosphorylated p125^FAK^	[57]
	Increased AGEs	[61]
	Increased O-GlcNAc glycosylation and decreased expression of Gal-7	[62]
	Downregulated the p38/MAPK pathway followed by inactivation of autophagy	[76]
	Increased percentage of M1 macrophage infiltration followed by increased secretion of TNF-α, which upregulates TIMP1 expression	[77]
	Increased expression of FOXO1 stimulates the expression of MMP-9	[7]
	Impaired expression of K16	[58]
	Increased expression of suppressor of cytokine signaling-3 (SOCS-3)	[85]
	Increased expression of Connexin 43	[86]
	Increased keratinocyte resistance to acetylcholine (Ach)	[91]
Chronic inflammation	Increased neutrophil and macrophage infiltration	[49]
	Increased pro-inflammatory cytokines (IL-1, IL-6, IL-8 and TNF-α)	[93]
	Activation of the TNF-α and TLR4 signaling pathway	[95]
	Decreased expression of Wnt family member 7A (wnt7a)	[97]
	Increased expression of MIP-2 and MCP1	[92]
	Decreased COX-1 expression and increased COX-2 expression	[98]
Chronic infection	Reduced diversity of microbiota and promoted biofilm formation	[48]
	Bacteria directly influenced keratinocytes (increasing apoptosis, diminishing keratinocyte migration and proliferation)	[103]
	Decreasing mRNA and protein levels of human β-defensins-2 (HBD-2) and HBD-3	[104]
	Decreased expression of mRNA and protein of cathelicidin	[107]
Impaired angiogenesis	Impaired the recruitment and migration of endothelial cells and EPCs	[112]
	Increased the expression of Thrombospondin-1 (TSP-1)	[114]
	Decreased the expression of vascular endothelial growth factor (VEGF)	[116]

## Data Availability

Not applicable.

## Abbreviations

MMP, matrix metalloproteinases; TIMPs, tissue inhibitors of MMPs; IL, interleukin; AGEs, advanced glycation end products; ROS, reactive oxygen species; O-GlcNAc, O-linked N-acetylglucosamine; MAPK, mitogen-activated protein kinase; Leucine-rich repeat LGI family member, LGI; TNF, tumor necrosis factor; TLR, Toll-like receptor; MIP, macrophage inflammatory protein; MCP, macrophage chemoattractant protein; EPCs, endothelial progenitor cells.
